# Salivation from Inhaling the Breath of a Salivated Patient

**Published:** 1828-08

**Authors:** 


2. *Salivation from inhaling the breath of a patient labouring under that affection.*—We have taken some pains to ascertain the correctness of the following information, and feel compelled to receive it as true.

Mr. George Howard, of this city, in the year 1826, was requested to shave one of his friends, who was at the time a patient of Dr. Barnes and profusely salivated. Mr. H. found his breath extremely offensive, but was compelled to inhale it for some time, as the operation was tedious. He was himself in perfect health, and subsequently took no medicine of any kind; but in three days, a sore mouth, a copious spitting, and a disagreeable breath, began to show themselves; and it was soon apparent that he laboured under a true and profuse salivation. Dr. B. saw him and found his breath highly mercurial. In a few days all the symptoms disappeared, and left him in his usual health.

I am aware, that a certain class of physiologists will insist that there is in this statement some mistake; but why should they? Rejecting all the accounts that were *formerly* published, relative to the discovery of mercury in the systems of those who had died after having recently taken its preperations, we have in our own times unquestionable evidence of their absorption; we have, also, conclusive proofs, that foreign substances are eliminated from the system, through the kidneys, skin and lungs. If mercury, which is slowly volatilized, at a heat below that of the human body, should be introduced into it, why may it not escape with the gaseous exhalations of the lungs; and if these, containing mercury, should be inhaled by another person, why may they not excite ptyalism? We know no reason why they should not; seeing that mercurial vapour when generated by heat in the laboratory, speedily salivates those who inhale it.

